# Dental Hints Department

**Published:** 1906-05

**Authors:** B. H. Teague

**Affiliations:** Aiken, S. C.


### DENTAL HINTS DEPARTMENT

Conducted by B. H. TEAGUE, D. D. S., Aiken, S. C., to whom all contributions to this department should be sent.

Decay is more common in the teeth in the upper jaw than in the lower.—*Headlight.*

---

The Alabama Dental Assembly has published its transactions in pamphlet form.

---

Read article on Some Diet Delusions, by Woods Hutchinson, M. D., in McClures Magazine for April.

---

To clean an impression tray of particles of modeling composition, hold under hot water and rub with a soft pine wood stick.

---

The teeth decay more rapidly in pregnancy and constitutional diseases, especially in those cases where the nervous system is involved.—*Headlight.*

---

A VARNISH FOR MODELS.—Models may be coated with a varnish made by dissolving white wax in spirits of turpentine. —W. H. ELLIS, *Dentist's Magazine.*

---

The National Dental Critic is a new dental journal published and edited by Dr. Emory A. Bryant. The first number hauls the National Dental Association over the coals. It is going to be a pungent member.

---

TO REMOVE TIN FROM PLATES.—Small particles of tin adhering to vulcanite plates can easily be removed by mixing mercury with enough alloy so it will not flow and rubbing it over the plate under fingers.—C. W. SIEFKILL, *Rolfe, Iowa.*

---

TO MAKE HARD PLASTER MODELS.—Mix the plaster of Paris with one-sixth of its volume of pulverized slaked lime. After the model is dried place it in a ten per cent solution of zinc sulphate until it is thoroughly saturated. Remove and dry.—*Dental Era.*

Crown Work, Platinum Bands.—One point I wish to bring out is the tolerance of the gums to platinum bands and bridges. The gums do not recede, but, on the contrary, embrace the band and are perfect in color and cleanness.—Harry F. Hamilton, *International Dental Journal.*

---

Partial Lower Impressions.—It is necessary to have as little plaster as possible on the outside with greater bulk on the inside of the teeth, so in removal the impression may be forced back into the mouth, thus preserving the integrity of the really important feature.—L. P. Haskell, *Dentists' Magazine.*

---

Shrinkage in Rubber During Vulcanizing.–The amount of shrinkage depends not alone on the time the rubber is subjected to the process of vulcanization, but also upon the temperature. The lower the temperature and steam pressure, the less the loss in shrinkage and the less the contraction in cooling. Low heat and long time also insures an improvement in the texture of the product.—Geo. B. Snow, *Brief.*

---

To Restrict Flow of Solder.—In soldering gold, when it is desired to restrict the flow of solder to certain areas, with a sharp lead pencil draw a line around the desired area; the solder will not flow past the line. This is especially valuable in filling cusps of seamless crowns, to prevent the solder flowing up the sides of the crown.—F. W. Franklin, *Western Dental Journal.*

---

Control of Acidity in the Mouth.—I have had made some very hard lozenges (tabloids) of bicarbonate of soda. I advise my patients to put one-quarter of a lozenge between the upper molars and the cheek on going to bed, changing from one side to the other alternately. The soda dissolves slowly all night and neutralizes the acid saliva.—E. J. Wetzel, *International Dental Journal.*

---

**Sycosis of the Beard.**

Use peroxide of hydrogen, then apply the following ointment:

R. Sulphur ................................1 ounce
Oil of rose ................................2 drops
White vaseline ..........................4 ounces
M. Sig.: Apply at night.

—Dr. T. H. Whitney, *Med. Brief.*

Removable Bridge Work.—The value of removable bridge work depends entirely upon the accuracy with which the work is done. The fittings must be as nearly perfect as it is possible to make them, or the work cannot be satisfactory.—F. A. Peeso, *Dentist's Magazine.*

Hemorrhage after Tooth Extraction.—Personally I am of the opinion that obstinate hemorrhage from alveoli after extraction can be most certainly controlled by means of carbolized resin and alum. I have never yet had a case when this happy combination has failed to do its work.—J. W. Taylor, *British Dental Journal.*

Clasps for Partial Dentures.—The best clasp for this work is made of wire. Using 18 gauge gold wire, an inch and three-quarters long, doubled with the pliers, not quite close together. Fit it to the labial surface and side, then bending the ends at right angles, to be embedded in the rubber.—L. P. Haskell, *Dentist's Magazine.*

A Combination Filling.—In the use of soft gold built into a layer of soft cement, followed by cohesive foil and, when subject to abrasion, finishing with gold platinum foil, the good qualities of each ingredient are magnified, and the faults of each minimized. The cement is adhesive, the soft gold gives close adaptation, the cohesive gold resists lateral stress in contour, and the alloy of gold and platinum resists abrasion.—Clyde Davis, *Western Dental Journal.*

Infected Wounds.—Fine results are obtained in the treatment of infected wounds with a mixture of pure carbolic acid so that the mixture feels like mre oil or chloroform when poured on the hand. The formula is carbolic acid 30 parts, camphor 60 parts, and alcohol to make 100. The mixture keeps perfectly if evaporation of the camphor is prevented.—*Digest.*

Administration of Gas.—Gas does not act well where liquor and stimulants have been taken. Some people have the mistaken idea that they must "brace up" to have a tooth out. When they come to me with too much of that I tell them to go away and sober up before they can have gas at my hands. Tobacco and liquor contra-indicate the use of gas.—Dr. Straight, *Dental Register.*

PREPARING SENSITIVE CAVITIES.—The first application of the burr can be made absolutely painless in the most highly sensitive cavity by simply taking ethyl chlorid on the burr point and bringing it quickly into contact with the tooth.—J. M. GALE, *British Dental Journal.*

---

AN EGYPTIAN TOOTH BRUSH.—The native Egyptians universally use, for cleaning their teeth, a twig of wood, frayed at one end to form a brush. The wood comes from a tree known in Arabic as Al-Arak and has been used by the natives for cleaning their teeth ever since the year 3000 B. C. It possesses rare medicinal properties which are very beneficial to the gums, and its natural aromatic odor makes its use most agreeable.—*Brief.*

---

CASTS FOR CLASPS.—A method which I use in partial gold cases is, get an accurate impression with plaster of Paris and when the model is being cast pour soft plaster into the teeth and put a flat pin into them, allow it to harden, and then pour the balance of the model. When it is hard the teeth may be readily removed from the model and the plate struck up and the teeth put back in position and the clasps fitted to them, so the adjustment is absolutely accurate.—DR. STODDARD, *International Dental Journal.*

---

DO NOT USE DIOXIDE OF HYDROGEN IN A MAXILLARY SINUS.—I have been asked whether I would use dioxide of hydrogen in a maxillary sinus. I would say most emphatically, no! In the first place, unless you have secured two large counter openings in the antrum, you will produce tremendous pressure by the liberation of the extra atom of oxygen and cause your patient a great deal of pain and suffering; consequently I say, no; do not use it.—M. L. RHEIN, NEW YORK, Dental Sum*mary.*

---

COST OF PROTECTING PATENTS.—"Patents cost too much," said an inventor. "It should be as cheap to patent an invention as it to copyright a book. It should cost but a dollar. As it is many a man hits on a good invention and then is afraid to risk his money patenting it. No wonder, either. Do you know what it costs really to protect an invention—to protect it all over the world? It costs $2,500, and it requires the taking out of no less than sixty-seven different patents."—*Newspaper.*

Handling Children.—I would rather prepare with hand instruments and fill with cement in five minutes and replace later, than remove nerve with broach and bur and consume an hour for a permanent filling in the mouth of a child. I know if I only work five minutes and cause no pain they will come back again, but if pain is caused I am not certain, and a child never takes a back seat to advertise us even in the most public places.—*C. Wesley Siefkin, D. D. S., Dental Brief.*

---

Don't Use Iodoform.—Don't use iodoform in root-dressings. Black's 1-2-3 mixture is much better, and is sweeter. Iodoform is only useful in surgery where granulation of wounded tissue is desired, and this condition does not pertain in the roots of teeth. The disagreeable results from the use of this drug are many.

I have a case on record where fifteen years of constant headache were caused by iodoform in the roots of a molar, and the malady was cured by removing the drug and properly treating the roots.—*C. P. Haselden, Brief.*

---

A Corner Inlay.—After matrix is invested and dried, puncture the matrix with instrument near cutting edge (which will be the heaviest part of inlay) large enough to push through a little way in an ordinary pin broken from vulcanite tooth. After the pin is in and left sticking up in your matrix proceed to build up and bake as usual. Before setting cut enough from tooth to allow room for pin and make an undercut. The inlay will never come out after set, unless broken.—*Dental Summary.*

---

Train Stopped by Losing Teeth.—The other day up in northeast Iowa one of the local trains running out of Sumner was stopped by the brakeman of the train in the endeavor to locate a set of artificial teeth that had become dislodged by some cause and rolled out into the right of way. The train was loaded with passengers and after train had stopped and backed up to the place where the teeth were said to be, the train crew and all of the passengers alighted from the train and began a systematic search for the missing denture. After a thorough search it was finally given up as lost forever. The engineer climbed back on the engine and rang the bell, the conductor called "all aboard" and the train moved once more.—*C. W. Darnell, D. D. S., Sumner, Iowa.*

Advantages of the Clasp.—There is an unjust prejudice in the minds of some patients, and also dentists, against the use of clasps. This is probably due to an improper knowledge of their advantages and disadvantages; also an insufficient knowledge of the mechanical principles involved and their practical application. The advantages of the clasp may be summed up in the statement that there is no method by which a partial plate denture can be retained with so much comfort and usefulness to the patient as with clasps. This implies that the conditions are favorable and that the method is properly applied.—*George H. Wilson, Dental Summary.*

---

Repairing Fractured Casts.—A valuable method of repairing plaster casts may be found in the use of celluloid dissolved in camphor and ether to a creamy consistency. A good quality of celluloid should be selected and to it should be added a mixture of equal parts of ether and spirits of camphor. This combination dissolves celluloid rapidly and should be added to the material until a solution of a creamy consistency is obtained. The preparation should be kept tightly corked to avoid its evaporation.

When it becomes desirable to repair broken casts the fragments to be attached should be well dried and both surfaces should be freed from broken particles. The surfaces should be coated with the celluloid solution, and after being pressed firmly should be allowed to dry.—*S. M. Weeks, International Dental Journal.*

---

Dental Hints Discontinues Publication.—The January issue of the *American Journal of Dental Science,* published at Madison, Wisconsin, announces that it has taken over the editor of *Dental Hints* and absorbed that journal. As a clearing house for disposing of superfluous dental journals Brother Beecroft has the record, he shouldn't let the *International* with its splendid record, lapse into oblivion without some formal ceremony.—*Editorial Register.*

It would be pleasing to the editor of the oldest dental journal to absorb both the quick and the dead.

---

Antiphlogistine.—Recently I had rather an interesting case which I thought some of the readers of your excellent publication might be interested in. I had set a bridge for a lady one of the abutments of which was a Richmond crown on a cuspid. The second day after, my patient called and complained of severe pain in the root of the crowned cuspid. An examination showed all the symptoms of an incipient abscess. The crown was slightly elongated and extremely sensitive to touch, with a sensitive spot opposite the apex of the root. The case was too sensitive to think of removing the crown, so I prescribed a thick poultice of "Antiphlogistine" (a preparation of fine clay mixed with antiseptic oils) on the side of her face over the tooth. The second day she called again and I found a decided improvement. The pain was all gone, but the tooth was still a little sore. The third day the soreness was all gone and the tooth felt as good as any other, and both patient and dentist were much pleased.—*Dr. F. H. Wilkinson, Brief.*

---

Death of President Lewis.—Mr. H. M. Lewis, the president of the S. S. White Dental Manufacturing Company, for many years, died January 26, 1906. Any one who knew Mr. Lewis will be very sorry to learn of his death. He was the great mind of that corporation, and one of the most capable business men in the dental supply business. He was universally honored and respected by all the dental trade and many of the profession. He was one of the most kindly and courteous gentleman we ever had the pleasure of meeting, and we cordially sympathize with his family and friends in their great loss.—*Editorial Register.*

In all the above we heartily concur.—Editor.

---

Pain After Extraction.—Thoroughly currett the alveolus, thus removing all disorganized tissue, coagulated blood, and alveolar debris, if any is present. The alveolus is then copiously irrigated with hot water, and after drying with cotton, an application is made of two drops of pure carbolic acid, and the alveolus is loosely packed with sterilized gauze. If the pain does not subside, an application should be made of campho-phenique and morphin-acetate. A small pellet of cotton is saturated with campho-phenique, and after taking up with it a small amount of morphin-acetate, is carried to the bottom of the painful alveolus. The morphin-campho-phenique dressing produces most remarkable results in the sense that the pain disappears almost instantaneously with the introduction of the dressing.—*Dr. Endelmann in Dental Register.*

---

Water as a Local Anaesthetic.—J. A. Wyeth has become convinced that the Schleich method of anæsthesia by the infiltration of weak cocain solutions is due more to the fluid itself than the contained salt. He gives the history of two cases, both men, with small tumors on the back. In both removal was painlessly done under water anæsthesia, which acts by pressure on the end organs of the sensory nerves, destroying their sensibility. Ten minims of sterile water were used, the needle resting in the substance of and not below the skin. This procedure was repeated in the same continued linear direction, until a sufficient length of anæsthetized skin was produced. The needle was thrust between the capsule of the tumor and the subcutaneous aureolar tissue and the water freely injected so as to lift the skin from the tumors, which was separated also by a similar manipulation from the underlying connective tissue.—*New York Medical Journal.*

---

Crowning Without Devitalizing.—It is occasionally possible to crown without devitalizing, as in malformation or malposition, as in case of peg laterals, erupted either in or out of the arch, where the peculiar shape of the exposed part of the crown is such as to require little if any mechanical preparation and where perhaps it would be inadvisable to employ a dowel crown because of the doubtful character of the root; a shell crown in combination with porcelain, known as the pulp preserver, being still protected from external influences by the enamel.—*Dr. Alden Bush, Summary.*

---

Gastralgia.—Gastralgia is one of the most painful conditions of the stomach with which we have to deal. It being almost next to impossible to relieve the paroxysms of excruciating pain. I have found the following prescription of great value in this trouble, if giving almost immediate relief, even in those cases where everything else had failed:

℞ Ol. Caryophyllus . . . . . . . . . . . . . . . . . .2 drachms.
Tr. Opii. Camph . . . . . . . . . . . . . . . . . . .2 ounces.
Spts. Vini Gallici . . q. s. ad. . . . . . . . . . .3 ounces.

M. Ft. Sig.: One drachm in hot water every two or three hours until relieved.—*Edwin A. Weimer, M. D., Med. Brief.*

---

A New Method of Producing Caoutchouc.—Two professors at the agricultural department of the University of Tokio have developed a new method for the production of caoutchouc from the bark of certain trees which grow abundantly in Japan. The high price of caoutchouc and the present method of producing it (destroying the trees) will favor this new industry in Japan in the near future if the new process of manipulation should prove satisfactory. It is claimed that according to this new process only the bark of the trees has to be used, without destroying the tree in its entirety, and that as a by-product large quantities of Japanese wax are obtained.—*Exchange.*

---

Extraction vs. Expansion.—Nature intends the full number of thirty-two teeth to tbe present in every mouth, this being in harmony with all her designs for the welfare of an individual. If no teeth have been lost, a proper expansion of the two arches will make them fit each other perfectly and a result giving the greatest utility as well as the greatest beauty is attained. Extraction interferes with nature's wise plans and nearly always makes this perfect result impossible. Aside from this, expansion of the dental arch is very much easier to accomplish than any proceeding in which extraction plays a part. Being both better and easier, expand, don't extract.—*Western Dental Journal.*

---

Sensitive Dentine.—I use desiccation, preceded by 95 percent carbolic acid in cases where the desiccation causes pain. I also use carbolic acid on a pellet of cotton, driving the agent into the dentin with a heated instrument applied to the cotton, and this followed by dryness. I use the Knowles obtunder with good effect in some cases, but most of all I use keenly sharp instruments, with the utmost delicacy of touch, and this latter, though very old and very hackneyed, is after all the best and most logical solution of the problem. If the profession generally would develop sufficient adroitness in the manipulation of instruments we would not hear so much about the terrors of sensitive dentine.—*Dr. C. N. Johnson, Cosmos.*

---

Excess in Eating.—A hint to those who may thoughtlessly at some time or other indulge in excess eating. If this indiscretion is committed, especially in highly seasoned things with rich sauces, a draught of cold water, acidulated with lemon juice or sulphuric acid, will take off the sense of weight at the stomach and assist the digestive process by moderating the alimentary fermentation. In this connection one may also say that one of the best remedies for obesity, and an exceedingly simple one, is to eat only one thing at a meal. It does not matter greatly what that one thing is, whether it is any one kind of fruit or any one kind of grain, but the prescription is: eat one article, and one article only, at one meal.—*Med. Fortnightly.*

---

GERMILETUM, THE IDEAL DENTIFRICE.

One of the prime requisites of an ideal dentifrice is that there shall be no acid reaction. The dental profession will find in germiletum a perfect antiseptic, slightly alkaline, with no acid reaction, fully meeting the delicate requirements in dental surgery, without any detrimental after effects of corroding acids, as no dentist would ever use any preparation that would in the least degree effect the enamel of the teeth. As a pleasant, efficient mouth wash, Germiletum is unexcelled, however, the best commendation is an impartial trial, therefore, for this purpose, the Dios Chemical Company will furnish to the dentists a full size bottle, together with several one ounce bottles for his patients, free, they paying only express charges.

---

Many dentists, although they use antiphlogistine in their families may not as yet have discovered how eefficacious it is when properly used in their dental practice. I have used it with highly satisfactory results in cases where there is alveolar inflammation. In cases of diseased antrum I have found antiphlogistine of the greatest value.—*Frank W. Brandow, D. D. S., Pittsfield, Mass.*

---

SUBDUING PLATINUM MATRIX.—Patinum should be annealed at least three times. First, before using; second, after first burnishing; third, preparatory to final treatment, which should consist of only packing spunk in the cavity. If any burnishing with instruments is attempted just prior to the final removal of the matrix it will be found that it will spring. This is especially true when dealing with compound cavities.—*Dr. W. A. Piper, Dominion Journal.*

---

TO ROUGHEN THE SURFACE OF A SOLID GOLD INLAY.—Take a plugger point that has been well tempered and grind the end to a needle point. Insert in the engine mallet, place the inlay in something solid, preferably a hammer handle with a small end so the inlay may be held firmly, and while running the engine at a high speed direct the blow at different angles upon the inlay. This produces a surface to which cement will adhere firmly.—*Dr. G. S. Hershey, Review.*

Pulpless Teeth and Neuralgia.—Teeth without living pulps are never the cause of neuralgia. All cases examined by Dr. G. V. Black indicate that there has never been one case resulting from teeth with pulp removed. When apparently otherwise, examinations have not been thorough and portions of living pulp were certainly present.—*Dr. James G. Atterberry, Western Dental Journal.*

---

Crawcraw in England.—Mr. Dencer Whittles, a lecturer on dental pathology at Birmingham University, is reported (says *Med. Record*) as saying that numerous cases of crawcraw have been introduced into that city. Several instances are described in which the infection appears to have been transmitted from one individual to another by kissing.

---

Physicians Should Examine Teeth.—No physician at the present time should ever treat a patient complaining of symptoms of indigestion without a thorough examination of the teeth. If those present are insufficient for proper mastication, or if there are carious teeth the patient should at once be recommended to consult a good dentist. It is not, however, sufficient simply to suggest any dentist, but the patient should be given a good idea of the risk involved in consulting anyone but some member of the dental profession who is thoroughly responsible and whose skill can be relied on to do what is best for the patient. There is no doubt that physicians can in this way contribute in an important degree to the uplifting of the sister profession of dentistry, and enable it to escape some of the demoralization incident to the large number of bargain-counter dentists in the field.—*Medical News.*

---

Ethics in Dentistry.—There is no line in dentistry that is more difficult to draw than the line that divides the ethical from the unethical man. The man, his surroundings, the customs prevailing in the profession at the time and the condition of the publc mind must all play a part. As we advance as a profession our ideas of what constitutes ethical practice will change to correspond. I wish to suggest that in each community at such a time every year, when our new graduates are entering upon their life work, members of our societies should call upon each one as early as possible and invite him to join in society work and sign the code of ethics before he has time to think of advertising. Get them before they take the wayward step and they will become an example in the community.—*Dr. Arthur D. Black, Review.*

To Hasten the Solution of Gutta Percha in Chloroform.—Chloropercha gets out of service now and then when it is much needed because of loss of chloroform through evaporation. To get it into shape again for immediate use, add the solvent and immerse the container in a dish of hot water. The chloroform begins to boil forthwith and the material is ready for immediate application.—*Office and Laboratory.*

---

Pyorrhoea Alveolaris: Common Salt.—In a case which had resisted treatment for a great many years, after the loss of many teeth a cure was affected through the use of common salt worked down into the pockets several times a day, salt being also used on the toothbrush. The gums became solid and healthy and recession stopped. The loose teeth became so firm that they were used as supports for a bridge.—*E. H. Allen, Dental Review.*

---

Dr. H. P. Carlton said that he had never yet written a paper of this character nor spoken his thoughts along this line, that the discussion did not at once turn to the question of courses and years. He wants to establish foundation courses and to leave the length of courses and curricula out of the question. He hopes to live to see it proved that the dentist of the future is going to be a medical man. The more a man gets in brain development the better dentist he will be. A man cannot be too broad and too scientifically trained to be a dentist.—*Journal American Medical Association.*

---

Sanitary Bridgework.—That a fixed bridge is never indicated I am not prepared to say, but that the removable bridge has a wider field, with greater possibilities and many advantages, I am thoroughly convinced. We have incomparably a more sanitary denture, greater possibilities of restoration of the lost tissues, whereby more natural and artistic results are obtained. Repairs are made easily and any subsequent repairs upon adjoining teeth are more simplified and less destruction of natural teeth is required for abutments. Another advantage of no small importance is the fact that good, serviceable and artistic bridges may be made with vulcanite at a moderate cost, but the ideal material for this class of work is porcelain.—*F. E. Roach, Review.*

---

What Was the Matter?—A dentist recently sent a patient, for whom he had inserted a full denture, to me to see if I could ascertain what was the reason her teeth did not work satisfactorily. I found by pressing the lower plate down to its bearings and removing the pressure the plate would be lifted up by the mass of glands and loose integuments, which would rise one-quarter of an inch above the margins of the jaw on the lingual side when the plate was out. This is a very common fault in lower dentures, and in such cases the margins of the plate should be cut away until it is no longer lifted. This was not the only difficulty. The articulation was faulty and this is another common fault. Having ascertained the cause of the trouble it was a simple matter to relieve the patient. —*L. P. Haskell, Review.*

---

New Trial Ordered in Terry Dental Case.—The Supreme Court of New Jersey has reversed the judgment of the Trenton city district court, in which Dr. J. Lee Terry of Trenton was adjudged not guilty of practicing dentistry without taking out a state license. Dr. Terry was sued in the district court by the state board of registration and examination in dentistry. Edward D. Duffield, assistant attorney general, represented the state and J. L. Conard appeared for Dr. Terry. The supreme court decision is as follows: "The judge charged that if the jury found that the defendant while practicing dentistry was doing so as a student of a regularly licensed dentist the verdict should be for the defendant. We think this charge was erroneous. There was evidence that the defendant was not an assistant, but rather the principal, and that the dental operations he performed were performed independently and on his own responsibility and not under the direct and immediate supervision of the alleged preceptors." The judgment is reversed and a new trial ordered.—*Trenton American.*

